# Successful Whole-Body Spectral CT with Intra-Osseous Iodine Injection

**DOI:** 10.5334/jbsr.2283

**Published:** 2020-09-14

**Authors:** Adrienne Coche, Etienne Danse, Emmanuel Coche

**Affiliations:** 1 Department of Radiology, Université Catholique de Louvain, Cliniques Universitaires St-Luc, Avenue Hippocrate, 10–1200 Brussels, BE

**Keywords:** Computed tomography, spectral CT, contrast media, emergency, intra-osseous injection

## Abstract

**Teaching point:** Intra-osseous access for contrast medium injection represents an alternative route for emergency CT in patients with compromised venous access.

## Case

A 36-year-old male was admitted in our emergency department for severe trauma and burn. Resuscitation was initiated at the site of the accident. The burned surface was evaluated as extensive (i.e., 80% of the skin surface). As intravenous access was impossible, two intraosseous needles were inserted, at the anterior and proximal right tibial diaphysis and the left humeral head.

**Figure 1:**
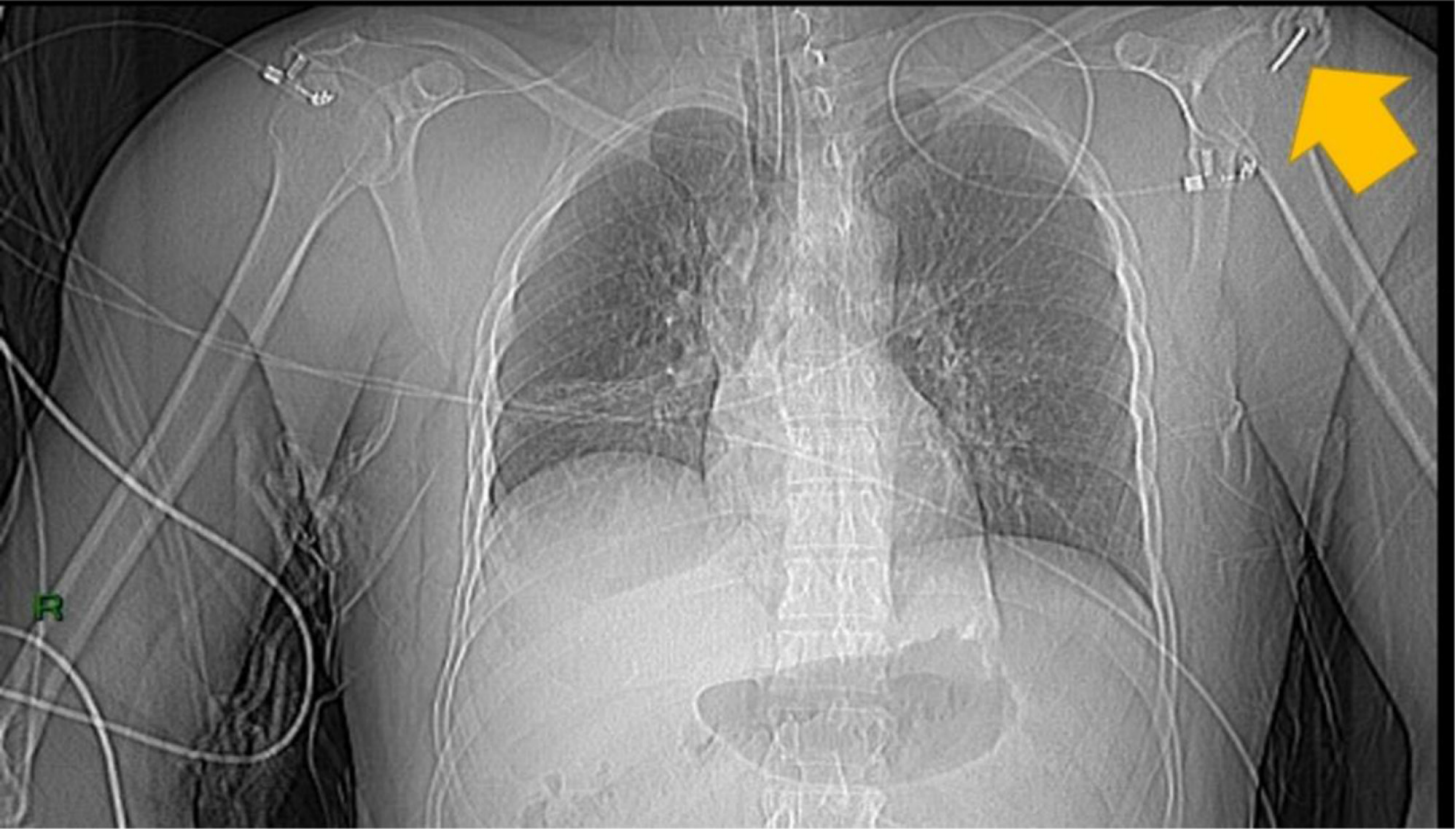


Whole-body computed tomography (CT) was performed on a dual-layer spectral CT (IQON, Philips Healthcare, Cleveland, OH, USA) at 120 kVp, 3 mm slice thickness. After unenhanced cranial CT, two thoraco-abdominal acquisitions were performed using the humeral head catheter for the iodine injection, as illustrated on the scout view (Figure 1, arrow).

**Figure 2:**
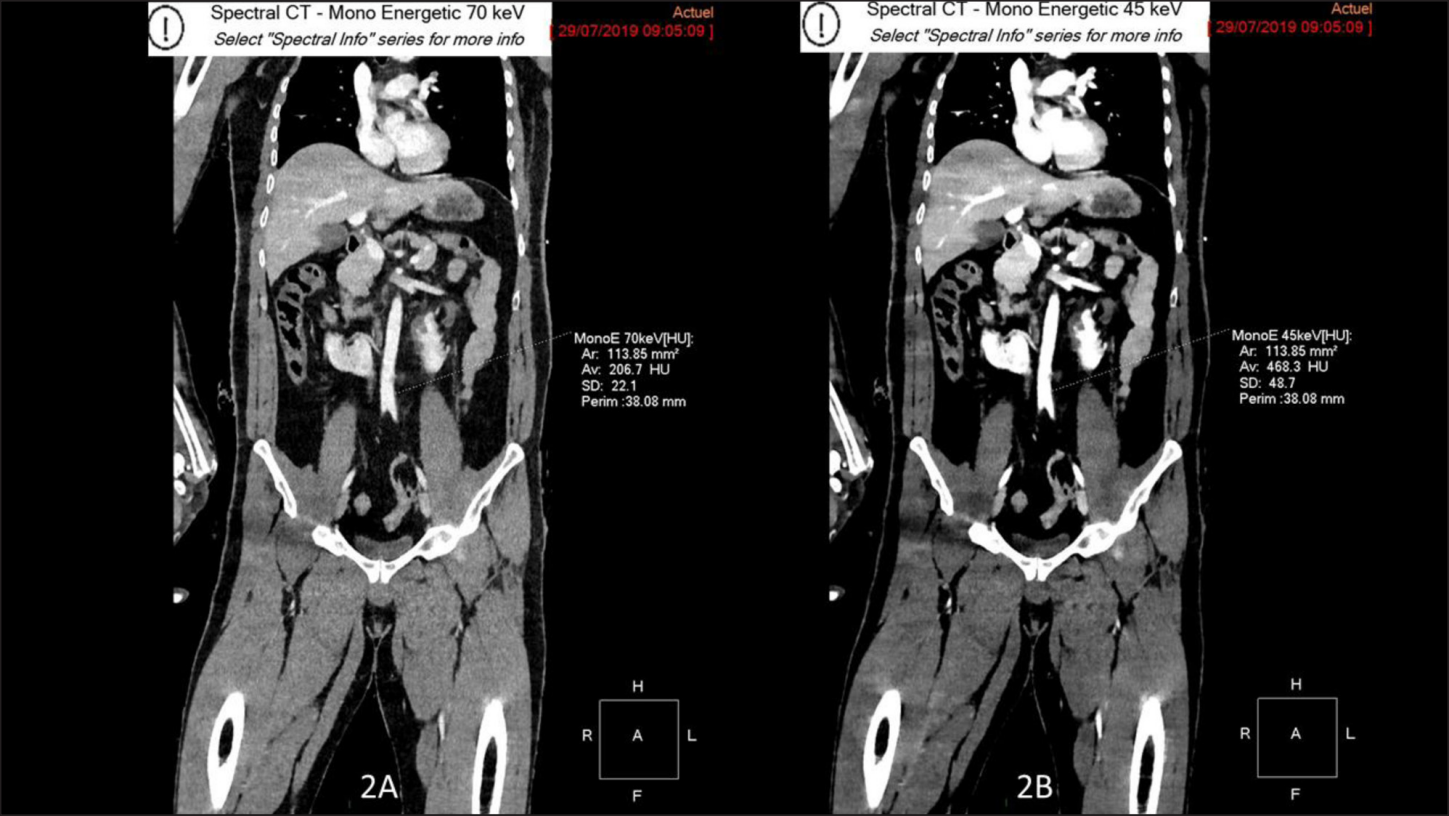


A first acquisition was performed at the arterial phase and a second one at the portal venous phase using 100 mL of Iobitridol (Xenetix 350, Guerbet, France) with a flow rate of 2ml/sec. A bolus tracking was used with a region of interest placed in the thoracic aorta and a start with a threshold at 100 UH. Frontal reconstructions were made using spectral-based images (SBI) at 70 keV (equivalent to 120 kVp) (Figure 2A) and at 45 keV (Figure 2B).

**Figure 3:**
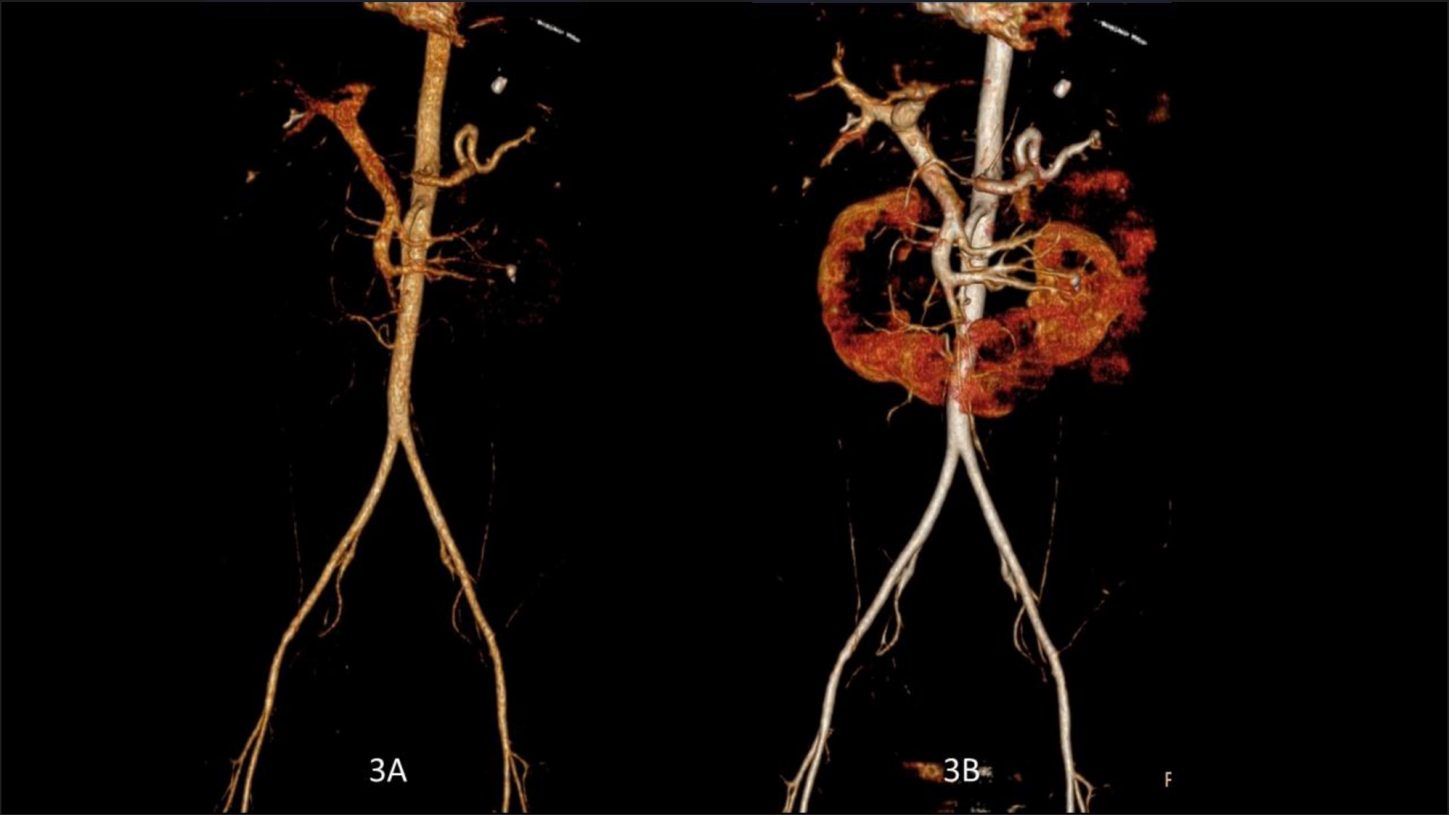


A good enhancement was obtained within the aorta. Intravascular enhancement was better shown at low keV. Three-dimensional reconstruction were performed at 70 keV (equivalent to 120 kVp) (Figure 3A) and at 45 keV (Figure 3B) to confirm the better visualisation of the small visceral arterial branches at low keV. There were no visceral lesions on the two series, but head CT revealed an acute subdural hematoma. The patient was immediately transported to the operating room and deceased within the two hours post-surgery, due to the extensive burned skin lesions.

## Comments

Bone marrow space has been used for many years as an alternative to conventional venous access in emergency situations, for the resuscitation and stabilization of pediatric and adult patients. Recently, Schindler et al. [1] evaluated the feasibility of intra-osseous contrast medium injection in emergency CT. There was a good-to-excellent image quality on chest and abdomen CT, without any significant difference as compared to acquisitions using intravenous contrast injection. Furthermore, when available, the use of spectral CT with virtual monochromatic images (low KeV) has the potential to enhance the intravascular density in the event of a suboptimal injection.
